# An Interpretable High-Accuracy Method for Rice Disease Detection Based on Multisource Data and Transfer Learning

**DOI:** 10.3390/plants12183273

**Published:** 2023-09-15

**Authors:** Jiaqi Li, Xinyan Zhao, Hening Xu, Liman Zhang, Boyu Xie, Jin Yan, Longchuang Zhang, Dongchen Fan, Lin Li

**Affiliations:** 1 China Agricultural University, Beijing 100083, China; ljiaqi0813@163.com (J.L.); zhaoxy@cau.edu.cn (X.Z.); xuhening@cau.edu.cn (H.X.); liman0711@cau.edu.cn (L.Z.); xieboyu@cau.edu.cn (B.X.); yanjin1213@cau.edu.cn (J.Y.); zhanglc@cau.edu.cn (L.Z.); 2 School of Computer Science and Engineering, Beihang University, Beijing 100191, China; 213352411@buaa.edu.cn

**Keywords:** rice disease detection, transfer learning, multimodality dataset, model interpreter

## Abstract

With the evolution of modern agriculture and precision farming, the efficient and accurate detection of crop diseases has emerged as a pivotal research focus. In this study, an interpretative high-precision rice disease detection method, integrating multisource data and transfer learning, is introduced. This approach harnesses diverse data types, including imagery, climatic conditions, and soil attributes, facilitating enriched information extraction and enhanced detection accuracy. The incorporation of transfer learning bestows the model with robust generalization capabilities, enabling rapid adaptation to varying agricultural environments. Moreover, the interpretability of the model ensures transparency in its decision-making processes, garnering trust for real-world applications. Experimental outcomes demonstrate superior performance of the proposed method on multiple datasets when juxtaposed against advanced deep learning models and traditional machine learning techniques. Collectively, this research offers a novel perspective and toolkit for agricultural disease detection, laying a solid foundation for the future advancement of agriculture.

## 1. Introduction

Rice, as one of the most crucial staple crops globally, directly sustains the dietary needs of billions. In Asia, particularly in East and Southeast Asia, rice is not only a primary food source but is also intricately linked with local culture, history, and economy [1]. However, like all crops, rice faces threats from a variety of diseases. These diseases, such as bacterial leaf blight, rice blast, and rice stripe disease, can lead to yield reductions and may also affect the quality and safety of the rice grains [2,3]. In traditional agricultural practices, farmers often rely on experience and intuitive observations to identify and manage these diseases. However, with the advent of modernized agriculture and precision farming, the efficient and accurate detection and prevention of crop diseases using contemporary technological means have emerged as focal points in agricultural research and technological development [4,5,6].

With the groundbreaking advancements of deep learning technologies in various domains, their potential in agricultural disease detection has also been progressively explored. Deep learning models, particularly convolutional neural networks (CNNs), have been demonstrated to excel in image classification and object detection tasks. In recent years, researchers have begun to deploy these models to identify and classify plant diseases. By analyzing images of damaged leaves, stems, and fruits [4,5,7,8,9], deep learning models can efficiently detect and locate diseases, thereby assisting farmers in taking timely countermeasures. Saleem et al. [10] employed deep learning meta-architectures like SSD, Faster RCNN, and RFCN to detect and classify plant diseases. Using the TensorFlow framework, they achieved the highest mAP of 73.07% with the SSD model optimized by Adam. Pal et al. [11] pioneered an innovative approach to plant disease detection by integrating image preprocessing to enhance image quality and employing a pretrained INC-VGGN model, which was further enhanced with a Kohonen learning layer to deepen feature understanding and improve classification accuracy. The work presented by Liang et al. [12] underscores the potential of computer-assisted approaches in the agriculture domain, showcasing the PD2SE-Net’s prowess in utilizing the multivariate characteristics of plant leaves to achieve superior classification performance, thereby enhancing the accuracy and efficiency of plant disease diagnosis systems. Leveraging the power of transfer learning, Chen et al. [13] paved the way for enhanced plant disease detection using deep convolutional neural networks. Their method, grounded in the use of pretrained models like VGGNet and the Inception module, exhibits exceptional accuracy and efficiency, marking a significant advancement in agricultural informatics. Jiang et al. [14] showcased a pioneering strategy in the agricultural informatics realm by integrating CNNs with SVM for enhanced rice leaf disease identification. Their rigorous methodology, which included optimizing SVM parameters through 10-fold cross-validation, resulted in significant improvements in recognition accuracy, indicating the promising potential of hybrid techniques in plant disease diagnostics.

Relying solely on visual information might not be sufficient to address all challenges. For instance, certain diseases might not be visibly evident in their early stages but manifest through chemical or molecular changes. This highlights the advantage of multimodal techniques, which can integrate various types of data—such as images, sound, vibrations, and chemical sensor data—to offer a comprehensive analysis of plant health. By consolidating different sensory information, multimodal techniques enhance the accuracy and reliability of agricultural disease detection, ensuring timely and precise identification and management of various diseases. In this context, the prominence of multisource data emerges. In agriculture, multisource data typically includes, but is not limited to, image data, climate data, and soil sensor data. Image data, often derived from drones or satellite remote sensing, provide valuable insights into vegetation health, growth status, and potential diseases. Various sensors, like soil moisture sensors, temperature and humidity sensors, and light sensors, furnish detailed and specific environmental parameters. While these data sources might have their limitations when used individually, their integration can present a holistic and three-dimensional profile of the agricultural environment. As outlined by Ouhami et al. [15], utilizing diverse sensor data, ranging from IoT devices to satellite imaging, combined with machine learning methodologies, has emerged as a potent strategy for detecting, predicting, and assessing these diseases. This comprehensive review underscores the evolving role of data fusion in disease detection, highlighting the potential of integrating information from varied sources to refine predictions related to plant health.

In a recent exploration of few-shot learning, a multimodal approach, vision-language pretraining (VLP), has been introduced, leveraging the relationship between different data modalities. Utilizing this, the study proposed an image-text-label-based recognition method for identifying cucumber diseases with limited samples, achieving a commendable 94.84% accuracy [16]. Zhou et al. [17] highlighted the shortcomings of image-only methods for crop disease identification, advocating for the integration of multimodal data. Focusing on diseases in tomatoes and cucumbers, they introduced ITK-Net, a model that synergizes “image-text” representation with domain knowledge, achieving high precision and specificity. Their work underscores the potential of melding domain knowledge with multimodal data for refined disease identification. Utilizing multimodal deep neural networks (DNNs), Trong et al. [18] presented an innovative weed classification technique in agriculture through a voting mechanism. Tested on the Plant Seedlings and CNU Weeds datasets with a range of DNN models, the method realized near-real-time image classification with peak accuracies reaching 98.77%. Furthermore, there is a wealth of research indicating that when different data sources are integrated, the accuracy and efficiency of crop disease detection can be significantly enhanced through the application of multimodal techniques. For example, in a recent study, deep learning was applied to UAV-acquired images to detect mildew disease in vineyards. Using both visible and infrared data, the method showed high accuracy in pinpointing diseased areas, offering a sustainable approach to vineyard management [19].

In summary, multimodal techniques offer an unprecedented opportunity in the agricultural domain, enabling more comprehensive and precise monitoring and prediction of crop health and potential diseases. The primary significance of utilizing multisource data lies in their ability to provide more accurate and detailed information. For instance, solely from image data, anomalies in vegetation might be identified, but pinpointing the exact cause could prove challenging. However, by integrating data from soil moisture sensors, it might be inferred that these anomalies resulted from overirrigation or water deficiency. Moreover, by incorporating temperature and humidity data, the probability of certain disease outbreaks can be predicted, facilitating proactive preventive measures. Nevertheless, the processing and fusion of multisource data present challenges. With different data sources potentially having varied formats, scales, and qualities, effectively aligning, cleaning, and integrating these data to bolster decision making emerges as a pivotal research issue. Against this backdrop, this paper proposes an interpretable, high-accuracy rice disease detection method based on multisource data and transfer learning. Comprehensive use was made of image data and various sensor data (such as soil moisture sensors, temperature and humidity sensors, light sensors, etc.), and through an innovative multisource data fusion module, high-accuracy disease detection was achieved. Additionally, a transfer learning strategy was introduced, enabling the model to exhibit robust generalization capabilities across different regions and environments. The primary contributions of this article span several key areas. First, an innovative data fusion module is developed to effectively integrate data from diverse origins and formats. This provides a more comprehensive and accurate informational basis for disease detection. Second, the model leverages a transfer learning strategy, enabling it to adapt to various environmental changes and thereby demonstrate strong generalization capabilities. Third, the model’s interpretability is enhanced through the design of an interpreter, which visually displays the reasoning behind the model’s decisions. This aids both farmers and researchers in gaining a better understanding of how the model functions. Finally, the efficacy and superiority of the proposed method are validated through extensive experiments conducted in multiple real-world scenarios.

## 2. Related Work

### 2.1. Application of Multimodality in Deep Learning

**Figure 1:**
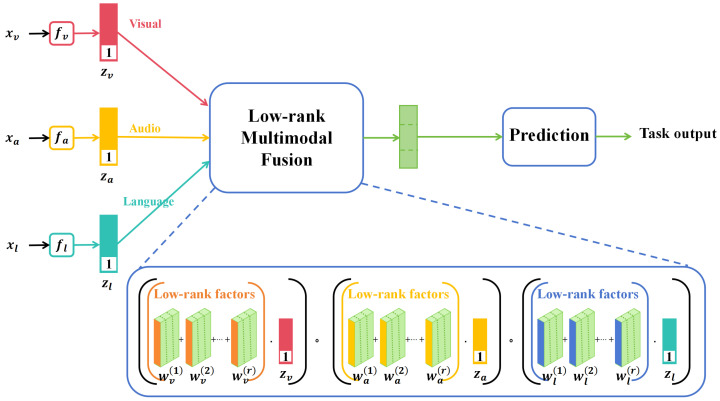
Illustration of the multimodality method [20] applied on image data, voice data, and text data.

In recent years, multimodal learning has garnered significant attention in the deep learning domain due to its ability to integrate information from diverse data sources, offering more comprehensive and accurate data representation. In various sectors such as agriculture, healthcare, and security surveillance, multimodal data have become commonplace, prompting focused research on effective data fusion methods, as shown in Figure 1.

In Figure 1, the term “low-rank” typically refers to a data matrix that, upon undergoing singular value decomposition (SVD) or similar techniques, reveals only a few nonzero singular values or eigenvalues. This suggests that the original high-dimensional data can be approximated by a significantly lower-dimensional space or features, thereby greatly simplifying both the model and computations. A low-rank structure often serves as an indicator of intrinsic correlations or patterns within the data. In the context of multimodal fusion, the low-rank assumption facilitates the extraction and integration of the most important and relevant information from diverse data sources, such as images and sensor data. Within this reduced-dimensional space, fusion algorithms, such as matrix factorization or multilayer perceptrons, are employed to combine features from different modalities into a unified representation. This fused low-dimensional feature set is then utilized to train the final deep learning model. Through this process, low-rank multimodal data fusion not only minimizes the dimensionality and computational complexity of the features but also captures relevant information from various data sources more effectively, thereby enhancing the performance of the final model.

A parallel strategy is first examined. In this approach, each data source is processed by an independent network structure, often referred to as subnetworks. Each subnetwork processes its corresponding modal data independently until a certain layer, where the outputs from these subnetworks are integrated. This integration can be a straightforward concatenation or a weighted average. Mathematically, this can be expressed as

(1) $$f\left(x_{1},x_{2},...,x_{n}\right){=\|}_{i=1}^{n}f_{i}\left(x_{i}\right)$$

where $f_{i}\left(x_{i}\right)$ denotes the network output of the *i*th data source, and ‖ signifies the integration operation. The primary advantage of this strategy lies in its simplicity and flexibility. As each modality has an independent subnetwork, it becomes feasible to select the most appropriate network structure for each modality. However, a notable drawback is the potential lack of adequate information interaction between different modalities, which may undermine the efficacy of data fusion.

Subsequently, an alternating strategy is explored. In this method, data from different modalities are processed alternately throughout the network, rather than being integrated at a specific layer. This implies that data from the first modality might initially pass through the early network layers, followed by data from the second modality, and so on. This strategy permits profound interaction and fusion of data from various modalities throughout the entire network. The main advantage of this approach is its ability to achieve deep data fusion, thereby effectively harnessing the correlation and complementarity between different modalities. However, a primary challenge of the alternating strategy is designing an efficient network structure to facilitate appropriate interaction levels between different modalities.

Lastly, a fusion strategy is discussed. Contrary to the previous strategies, the fusion strategy conducts explicit data fusion at a specific layer within the network. This is typically achieved through dedicated fusion modules, such as weighted summation, multiplication, or other fusion techniques. Mathematically, this can be denoted as

(2) $$f\left(x_{1},x_{2}\right)=w_{1}\cdot f_{1}\left(x_{1}\right)+w_{2}\cdot f_{2}\left(x_{2}\right)$$

where $w_{1}$ and $w_{2}$ are weights representing the importance of different data sources. The primary advantage of the fusion strategy is its capability to conduct data fusion at a definitive layer, providing a more intuitive understanding and control over the fusion process. Additionally, since the fusion occurs at a distinct layer, model debugging and optimization become more manageable. However, a significant challenge associated with the fusion strategy remains: designing effective fusion modules is still an open question.

In summary, multimodal learning has found widespread applications in deep learning. This study introduces a novel fusion strategy aimed at more effectively integrating information from different data sources, thereby achieving high-precision rice disease detection.

### 2.2. Interpretability in Machine Learning

With the growing complexity of deep learning and machine learning models, understanding their decision-making processes has become increasingly challenging. Especially in sectors that prioritize accuracy and reliability, such as medical diagnostics and financial risk assessment, the decisions made by a model must be lucidly explained and verified. Hence, enhancing the interpretability of machine learning models has emerged as a prominent research direction.

Activation mapping methods are initially considered. For convolutional neural networks (CNNs), understanding how the model recognizes and responds to specific features in the input data can be achieved by observing activations across various layers. Particularly in the final layers, activation maps can reveal the basis for the model’s decisions. Mathematically, for a given input *X*, the activation map at layer *i* can be expressed as

(3) $$A_{ij}=\sum_{k=1}^{K}W_{ijk}\cdot X_{k}$$

where $A_{ij}$ is the activation value of the *j*th neuron in the *i*th layer, and $W_{ijk}$ is the corresponding weight. The primary advantage of this method is its intuitiveness and simplicity, offering a clear insight into the role of each feature in decision making. However, for deeply layered or complex models, activation maps might not provide meaningful interpretations.

Feature importance methods are then examined. By analyzing model weights or employing techniques like SHAP or LIME, crucial features influencing model decisions can be identified. For decision trees or random forests, feature importance can be ascertained by observing the frequency of a feature’s usage across nodes or the information gain it provides when utilized. For deep learning models, feature importance is typically determined through model weights or specific interpretative techniques. Mathematically, for a given input *X*, the importance of feature $x_{i}$ can be defined as

(4) $$I\left(x_{i}\right)=\sum_{j=1}^{J}\left|W_{ij}\right|$$

where $I(x_{i})$ indicates the importance of feature $x_{i}$, and $W_{ij}$ is the weight from feature $x_{i}$ to the *j*th neuron. The main advantage of feature importance methods lies in providing a clear, quantitative mechanism for feature evaluation. However, its drawback is the potential inaccuracy or ambiguity in determining feature importance for certain models.

Lastly, decision tree and rule methods are discussed. The core idea behind this approach is to transform the decision-making process of deep models into a series of rules or decision trees, rendering them more comprehensible. For instance, for a classification problem, model decisions can be represented as a set of "if–then" rules. Mathematically, this can be described as

(5) $$y=\left\{\begin{array}{ll} c_{1} & \mathrm{if}\;R_{1}\\ c_{2} & \mathrm{if}\;R_{2}\\ \vdots & \vdots \\ c_{n} & \mathrm{if}\;R_{n} \end{array}\right.$$

where $R_{i}$ is a decision rule, and $c_{i}$ is the corresponding classification result. The primary advantage of this method is its clarity and directness, as the contribution of each rule to the decision can be directly observed. However, a significant drawback is that for highly complex models, the conversion to decision trees or rules might result in information loss or yield an overwhelming number of rules, making management and interpretation challenging.

### 2.3. Transfer Learning

**Figure 2:**
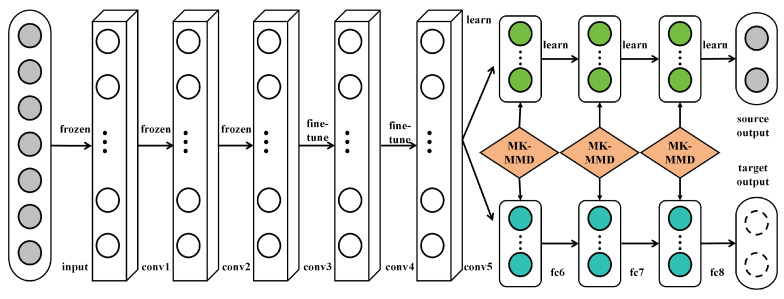
Illustration of the transfer learning mechanism used on AlexNet [21].

Transfer learning is a machine learning technique designed to apply knowledge acquired from one task to a different but related task, aiming to enhance the learning efficiency and performance of the latter, as shown in Figure 2.

In the context of deep learning, transfer learning typically involves utilizing a model pretrained on a large dataset (like ImageNet) as a foundation, followed by fine-tuning the model to cater to a specific target task. The underlying principle of transfer learning is rooted in the observation that many tasks share common low-level features. For instance, various image recognition tasks might require edge detectors or texture identifiers. Therefore, through transfer learning, starting model training from scratch can be avoided, saving substantial time and computational resources. Mathematically, given a source task *S* and a target task *T*, the objective of transfer learning is to leverage the knowledge learned from *S* to aid the learning of *T*. In deep learning, this is usually achieved through

(6) $$\phi \left(X\right)=F_{S}\left(X;\theta_{S}\right)$$

where $F_{S}$ represents the model learned on task *S*, and $\theta_{S}$ are its parameters. The model, pretrained on task *S*, is then further trained on data from task *T*. In this context, the model parameters commence from $\theta_{S}$, followed by fine-tuning:

(7) $$\theta_{T}=\theta_{S}+\Delta \theta$$

where Δθ is the parameter update obtained through training on data from task *T*.

Transfer learning has a wide range of applications. In the field of computer vision, it is frequently employed for tasks with limited labeled data, such as medical image analysis or fine-grained species identification. In natural language processing, pretrained embeddings like Word2Vec or BERT serve as instances of transfer learning, enhancing the performance of tasks like text classification or named entity recognition. The primary advantages of transfer learning are its efficiency and effectiveness. In scenarios with limited data for the target task, transfer learning can harness the data from the source task to boost model performance. Additionally, it can expedite model training as many crucial features have already been learned in the source task. In this study, the method of transfer learning, combined with multimodal data, is employed to provide an efficient and accurate solution for rice disease detection.

## 3. Materials and Method

### 3.1. Dataset Collection

#### 3.1.1. Image Dataset

**Figure 3:**
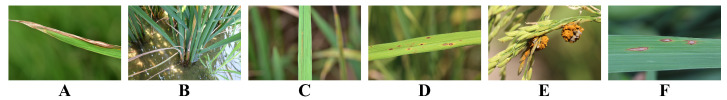
Illustration of the image dataset used in this paper. (**A**) represents white leaf blight; (**B**) represents brown spot disease; (**C**) represents bacterial stripe disease; (**D**) represents sesame leaf spot disease; (**E**) represents rice aspergillosis disease; (**F**) represents rice blast disease.

The dataset for this research primarily originates from two channels: a portion was collected in situ from the West Campus Science Park of China Agricultural University, and the remainder was obtained by web scraping. Due to its unique geographical location and climatic conditions, the West Campus Science Park of China Agricultural University provides an ideal environment for rice cultivation. In this area, five prevalent rice diseases were selected for collection, namely, white leaf blight, brown spot disease, bacterial stripe disease, sesame leaf spot disease, rice aspergillosis disease, and rice blast disease. The rationale behind selecting these six diseases is their widespread distribution in China’s rice-producing regions and their significant impact on rice growth and yield, making their accurate detection and classification of paramount practical significance. Data collection was conducted between September and November in the fall, a period when rice diseases are most prevalent, facilitating the acquisition of genuine and typical disease features. The collection device employed was the Canon EOS 5D Mark IV DSLR camera, achieving a resolution of 30.4 megapixels, ensuring that the collected images are of high clarity and rich in detail, as shown in Figure 3.

To further enrich the dataset and enhance the model’s generalization capability, related rice disease images were also scraped from the Internet. Using Python’s Scrapy and BeautifulSoup libraries, targeted image scraping was executed from mainstream search engines like Baidu Images and Google Images. These web-scraped images offer representations of rice diseases from various regions, lighting conditions, and angles, contributing to the model’s robustness in practical applications. The evident advantage of integrating both in situ collection and web scraping is clear. While in situ collection guarantees the authenticity and consistency of the data, web scraping broadens the data’s diversity and scope, enabling the model to tackle more complex and diverse scenarios. The specific distribution of the dataset is shown in Table 1:

#### 3.1.2. Sensor Dataset

In addition to image data, relevant sensor data were also collected. Sensors utilized include temperature sensors, humidity sensors, and soil moisture sensors. These sensor data provide detailed information about the rice growing environment, such as climatic conditions and soil humidity, which are critical factors influencing the onset of rice diseases.

### 3.2. Dataset Preprocessing

#### 3.2.1. Image Data Augmentation

In deep learning and machine learning, data augmentation is a prevalent technique employed to expand the training dataset, thereby enhancing the model’s generalization ability. For tasks like agricultural disease detection, data augmentation can not only aid the model in better coping with various changes in real-world scenarios but can also reduce the risk of model overfitting, especially when training data are limited. There are many methods for data augmentation, with the most common ones including:

1. Rotation: By randomly rotating the image by a certain angle, the model’s robustness to variations in object orientation can be enhanced. Mathematically, given an image *I*, its rotated version can be represented as
  (8) $$I^{\prime }=R\left(I,\theta \right)$$
  where *R* represents the rotation operation, and θ is the rotation angle.
2. Translation: Randomly shifting the image position helps the model learn about object positional variations. Mathematically, the translation operation can be expressed as
  (9) $$I^{\prime }=T\left(I,\Delta x,\Delta y\right)$$
  where *T* signifies the translation operation, and Δx and Δy are the translation distances in the x and y directions, respectively.
3. Scaling: Altering the image size allows the model to better recognize objects of different sizes. Mathematically, the scaling operation can be described as
  (10) $$I^{\prime }=S\left(I,\alpha \right)$$
  where *S* is the scaling operation, and α is the scaling factor.
4. Mirror Flipping: A simple yet highly effective data augmentation method that helps the model learn about different object orientations. For instance, for horizontal flipping, the operation can be represented as
  (11) $$I^{\prime }=F_{h}\left(I\right)$$
  where $F_{h}$ indicates the horizontal flipping operation.

**Figure 4:**
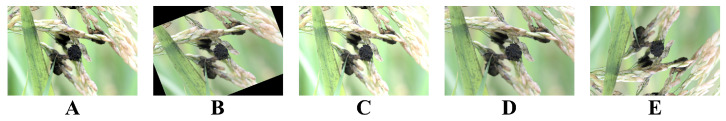
Illustration of the different image augmentation methods used in this paper. (**A**) represents contrast augmentation; (**B**) represents rotation augmentation; (**C**) represents brightness augmentation; (**D**) represents cropping augmentation; (**E**) represents flipping augmentation.

These augmentation methods can be used independently or in combination, generating a plethora of training samples, as shown in Figure 4.

#### 3.2.2. Multisource Data Alignment

In multimodal learning, data alignment is a pivotal step, ensuring that information from different data sources can be correctly integrated. For this research, it is imperative to align the image data and sensor data, ensuring their consistency in both time and space. Firstly, for time alignment, suppose there is an image dataset $\left\{I_{t}\right\}$ and a sensor dataset $\left\{S_{t}\right\}$, where *t* is the timestamp. For each $I_{t}$, a corresponding $S_{t}$ needs to be found such that the difference in their timestamps is minimal. Mathematically, this can be represented as

(12) $${\hat{S}}_{t}=\arg\min_{S_{t}}\left|t-t^{\prime }\right|$$

where $t^{\prime }$ is the timestamp of $S_{t}$. For spatial alignment, it is crucial to ensure that the image data and sensor data originate from the same or proximate locations. This is typically achieved through GPS coordinates. Assuming each image datum $I_{x,y}$ has a corresponding GPS coordinate (x,y), a sensor datum $S_{x^{\prime },y^{\prime }}$ needs to be identified such that their coordinate differences are minimal. Mathematically, this can be expressed as

(13) $${\hat{S}}_{x,y}=\arg\min_{S_{x^{\prime },y^{\prime }}}\sqrt{{\left(x-x^{\prime }\right)}^{2}+{\left(y-y^{\prime }\right)}^{2}}$$

Through the aforementioned time and space alignment methods, consistency of multisource data in both time and space can be ensured, laying a solid foundation for subsequent multimodal learning.

### 3.3. Proposed Method

#### 3.3.1. Overall

**Figure 5:**
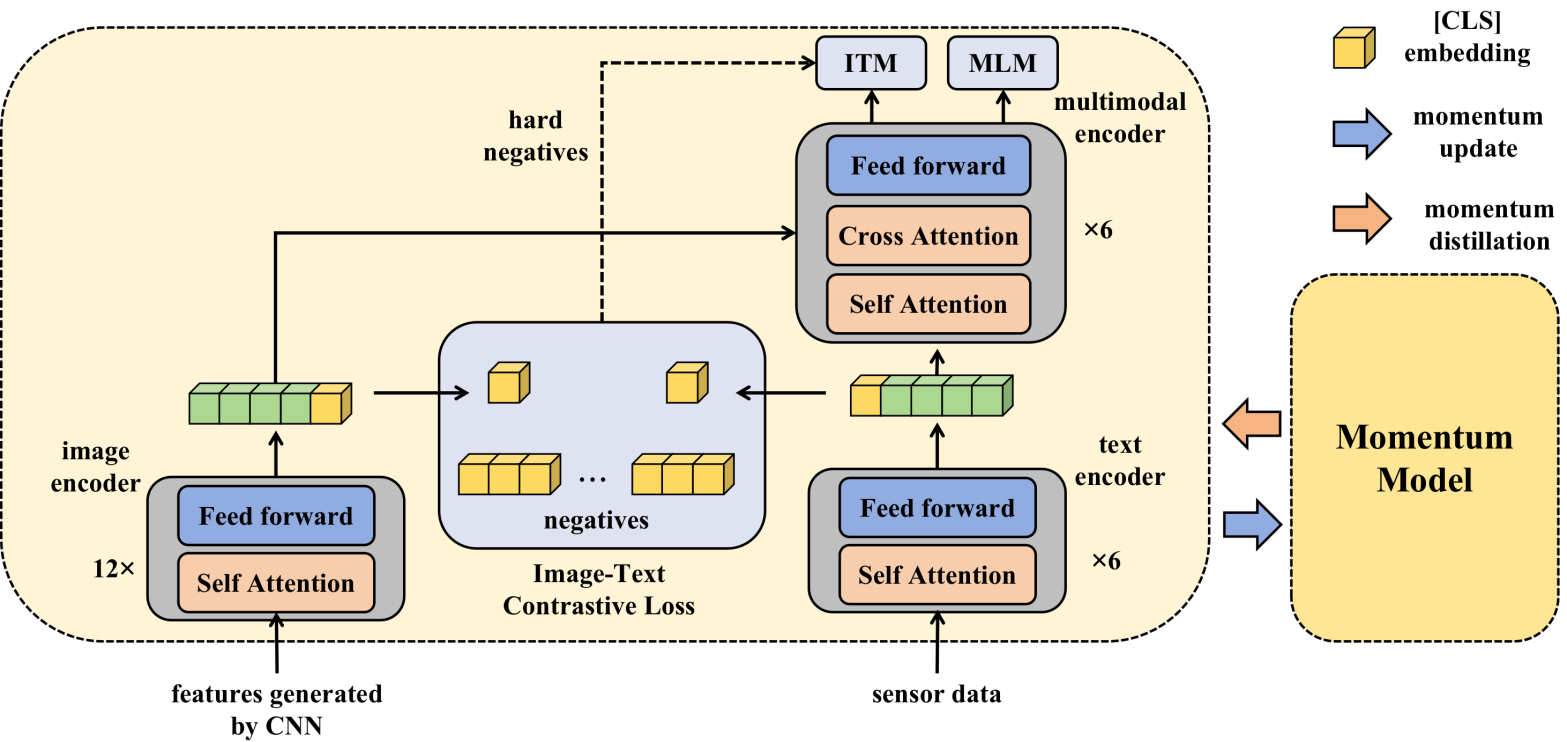
Illustration of the whole method proposed in this paper.

In the context of agricultural disease detection, a deep learning framework, based on multimodal data and transfer learning, is proposed. This framework incorporates a model interpreter to enhance the transparency and credibility of the model. To comprehensively capture the health status of rice, data from different sources are initially integrated. Each modality, whether it is image data or sensor data, harbors unique information about the condition of the rice. As a result, a specific fusion mechanism was designed that allows each modality of data to be independently processed through its specific subnetwork during the initial stages, the image features inputted into the Transformer structure in Figure 5 are preprocessed by a CNN model, and then it integrates these features at deeper levels to form a unified feature representation. This representation captures the complementary information between modalities, providing a richer context for the subsequent classification task, as shown in Figure 5.

To maximize the utilization of existing knowledge and accelerate the training process, transfer learning strategies were incorporated. Deep models, pretrained on large datasets, were chosen as the initial structure, leveraging their already acquired rich features. These models were then fine-tuned to better adapt to the specific task of rice disease detection. This transfer learning strategy, combined with multimodal data, not only significantly improves the training efficiency of the model but also ensures higher accuracy. However, the decision-making process of deep learning models is often challenging to comprehend, which could lead to skepticism from farmers and agricultural researchers regarding the model’s decisions. To address this issue, a model interpreter was further designed, with the primary goal of revealing the decision-making logic of the model. By delving into the model’s intermediate layer activations, feature weights, and feature maps, the interpreter provides a clear and intuitive explanation for every decision made by the model.

By integrating these three core components, the methodological framework proposed in this study demonstrates its formidable capability. The multimodal data fusion strategy ensures comprehensive data input; the transfer learning strategy delivers an efficient training process; and the model interpreter guarantees decision transparency and credibility. More importantly, there exists profound interconnection and complementarity among these three parts. The fusion of multimodal data provides a richer feature foundation for transfer learning, and the model obtained through transfer learning, owing to its enhanced performance, becomes more interpretable by the model interpreter. This tight integration ensures exceptional performance of the entire framework in rice disease detection, offering new perspectives and tools for future agricultural research and applications.

#### 3.3.2. Multisource Data Alignment Module

**Figure 6:**
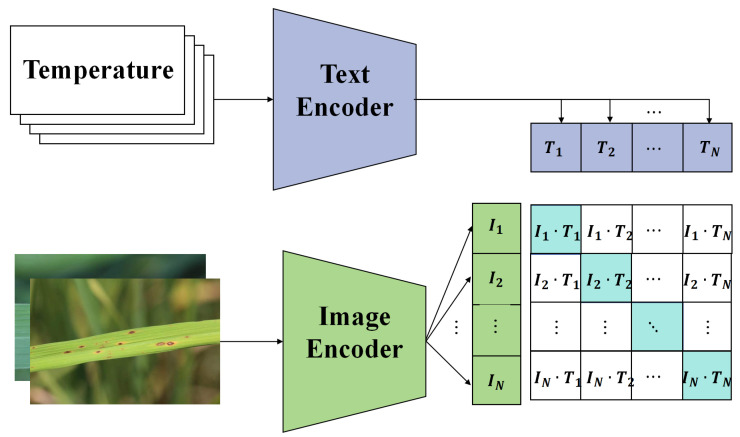
Illustration of the multisource data alignment module structure.

In multimodal learning, the fusion of information from different data sources presents a critical challenge. Distinct data sources might have varying distributions, scales, and features. Direct fusion could lead to information loss or the introduction of noise. To effectively integrate these data while ensuring the integrity of information, a multisource data alignment module is proposed, as shown in Figure 6.

Recognizing that there are two primary data sources, image data and sensor data, features are first extracted from both. Let the feature representation of the image data be denoted as $f_{img}\left(X_{img}\right)$ and that of the sensor data as $f_{sen}\left(X_{sen}\right)$, where $X_{img}$ and $X_{sen}$ represent the input for image data and sensor data, respectively. To achieve data alignment, a transformation function *T* was designed such that

(14) $$T\left(f_{img}\left(X_{img}\right)\right)\approx f_{sen}\left(X_{sen}\right)$$

The goal of *T* is to identify a mapping where the feature representation of image data, after transformation, closely aligns with the feature representation of sensor data. Mathematically, the objective of this alignment module can be expressed as

(15) $$\min_{T}{∥T\left(f_{img}\left(X_{img}\right)\right)-f_{sen}\left(X_{sen}\right)∥}^{2}$$

Gradient descent was employed to solve the aforementioned optimization problem. At each step, the parameters of the transformation function *T* are updated to gradually minimize the discrepancy between the feature representations of the two data types. Such a design offers several advantages:

1. Feature alignment: By introducing the transformation function *T*, features from different data sources are ensured to align in the same space, facilitating subsequent fusion operations.
2. Reduction in information loss: Direct fusion might result in information loss; however, through the alignment operation, the critical information from each data type is retained.
3. Enhanced model robustness: Models could be influenced by noise when data come from different distributions. The alignment operation mitigates this influence, bolstering model robustness.
4. Improved fusion efficacy: Data aligned in this manner are more amenable to fusion, potentially enhancing the overall performance of the model.

In summary, the multisource data alignment module serves as a pivotal component within the proposed framework. It ensures that information from different data sources is effectively integrated, offering robust support for subsequent classification tasks.

#### 3.3.3. Model Interpreter

Deep learning models, particularly convolutional neural networks (CNNs), due to their multilayered and intricate structure, are often perceived as “black boxes”. Despite their outstanding performance on various tasks, their decision-making process remains elusive. Addressing this issue, a model interpreter is proposed in this study, aimed at visualizing and elucidating the decision mechanisms of deep learning models, especially within the context of multimodal rice disease detection tasks. Initially, a feature map extraction mechanism was designed. Considering that convolutional layers are crucial for capturing local features in images, feature maps were extracted from each convolutional layer. For a given input image *X*, its feature map at the *i*th convolutional layer is

(16) $$F_{i}\left(X\right)=C_{i}\left(X;\theta_{i}\right)$$

where $C_{i}$ is the *i*th convolutional layer, and $\theta_{i}$ represents its parameters. To provide a more intuitive interpretation of these feature maps, an activation mapping mechanism was further developed. Activation maps are derived by applying activation functions (e.g., ReLU) to the feature maps, highlighting the spatial distribution of specific features in the original image. Mathematically, the activation map is

(17) $$A_{i}\left(X\right)=\sigma \left(F_{i}\left(X\right)\right)$$

where σ denotes the activation function. By examining these activation maps, regions of the image that the model focuses on during decision making can be visually discerned, aiding in understanding how the model identifies rice diseases. In addition to visualizing feature maps, a weight interpretation mechanism was devised. Within deep models, the weight of each neuron determines the importance of its corresponding feature. To elucidate these weights, a weight-ranking algorithm was introduced. This algorithm sorts all neuron weights based on their absolute values, thereby identifying the most crucial features. Mathematically, weight ranking is

(18) R(X)=sort(|θ|)

where *R* represents the outcome of weight ranking. The primary advantage of this design is its provision of intuitive and precise explanations for deep learning models. By observing feature maps and weights, users can gain deeper insights into the model’s decision-making mechanism, crucial for enhancing model trustworthiness and acceptance. In the context of multimodal rice disease detection tasks, this interpreter offers several benefits:

1. Enhanced trustworthiness: Agricultural researchers and practitioners can intuitively comprehend the model’s decision-making process via the interpreter, fostering greater trust in model outcomes.
2. Insight provision: The interpreter reveals image regions and pivotal features that the model focuses on, offering researchers novel insights into rice diseases.
3. Facilitation of model improvement: Through the interpreter, potential model shortcomings and deficiencies can be identified, paving the way for further model refinement and optimization.

In summary, the designed model interpreter offers lucid, intuitive explanations for deep learning models. Especially in the intricate task of multimodal rice disease detection, it plays a pivotal role in bolstering the model’s credibility and acceptance.

### 3.4. Evaluation Index

In machine and deep learning tasks, evaluation metrics serve as a pivotal measure for assessing the performance of models. The correct selection and understanding of these metrics are vital for ensuring the efficacy and reliability of models. In this study, to holistically evaluate the performance of the proposed model, precision, recall, and accuracy were chosen as the primary evaluation metrics. The mathematical definitions and the significance of these metrics in model evaluation will be elaborated upon below.

Precision is the ratio of samples correctly classified as positive to all samples predicted as positive by the model. Mathematically, precision can be defined as

(19) $$\mathrm{Precision}=\frac{TP}{TP+FP}$$

Here, TP stands for true positives, which represents the number of samples correctly classified as positive, while FP denotes false positives, which are the samples wrongly classified as positive. Precision reflects how many of the samples predicted as positive by the model are genuinely positive. A model with high precision indicates its high accuracy in predicting positive cases, minimizing the possibility of false alarms.

Recall, on the other hand, is the ratio of samples correctly classified as positive to all actual positive samples. Mathematically, recall can be defined as

(20) $$\mathrm{Recall}=\frac{TP}{TP+FN}$$

In this equation, TP stands for true positives, while FN represents false negatives, which are the samples wrongly classified as negative. Recall illustrates how many of the actual positive samples are correctly predicted as positive by the model. A high recall indicates the model’s capability to identify most of the positive samples, minimizing missed cases.

Accuracy provides an overall measure of the model’s performance by calculating the ratio of all correctly classified samples to all samples. Mathematically, accuracy can be defined as

(21) $$\mathrm{Accuracy}=\frac{TP+TN}{TP+TN+FP+FN}$$

Here, TP stands for true positives, TN represents true negatives, which are the samples correctly classified as negative, FP denotes false positives, and FN indicates false negatives. An elevated accuracy implies that the model performs well across all classification tasks.

Each of these metrics holds unique significance in model evaluation. While precision reflects the accuracy of the model in predicting positive cases, recall describes the model’s coverage capability for positive samples. Accuracy, in contrast, offers a comprehensive view of the model’s performance. By evaluating a model based on precision, recall, and accuracy, its reliability and effectiveness in real-world applications can be ensured.

### 3.5. Experiment Designs

To validate the effectiveness of the proposed method on multimodal data learning and model interpreters, a thorough and comprehensive experimental design was laid out. The initial challenge in the experimental phase was the rational partitioning of the data. To ensure the generalizability of the model and prevent overfitting, the dataset was split into training and validation sets. Specifically, 80% of the dataset was utilized for model training, while the remaining 20% served as the validation set for model verification and parameter tuning. To further ensure the robustness and reliability of the experimental results, a strategy of five-fold cross-validation was adopted. This implies that the data would be segmented into five parts, with one part used as the validation set in each iteration, while the rest would serve as the training set, ensuring a robust evaluation of model performance.

Subsequently, for model comparison and assessment, several classical deep learning models, including AlexNet [21], SVM [22], ResNet [23], GoogLeNet [24], DenseNet [25], VGG [26], and EfficientNet [27], were chosen as baselines for comparison. The rationale behind selecting these models as baselines is that historically, they have represented state-of-the-art techniques at various phases and have demonstrated exceptional performance across a range of image classification tasks. Additionally, spanning multiple stages in the evolution of deep learning, these models each possess distinct network structures and characteristics, facilitating a comprehensive evaluation of the superiority of the proposed method. During the model training phase, the choice of optimizer and the setting of hyperparameters are of paramount importance. In all experiments, the Adam optimizer was chosen for model optimization due to its superior convergence speed and performance in various deep learning tasks. For the learning rate, an initial value of 0.001 was employed, paired with a decay strategy that reduces the learning rate to 90% of its value every 10 epochs. Moreover, a batch size of 32 and a total of 50 epochs were set. These hyperparameters were based on preliminary experiments on the training set and recommendations from the related literature.

Lastly, to delve deeper into the contributions of each component of the proposed method, ablation studies were conducted. The aim of these studies was to exclude certain modules or features one by one and observe their specific impact on model performance. This would provide clarity on the necessity and role of these components. For instance, experiments were conducted by removing the multimodal data alignment module and solely training on unimodal data. Consideration was also given to eliminating the model interpreter, relying solely on the raw model output for decision making, and evaluating the performance of a model trained without the transfer learning strategy. Such ablation studies are integral to validating that each part of the proposed method actively contributes to the final model performance.

## 4. Results and Discussion

### 4.1. Detection Results

The aim of the experimental design was to compare the performance of different models on an image dataset. By evaluating the three core metrics, precision, recall, and accuracy, a comprehensive assessment of each model’s performance in disease detection tasks was achieved. The results are presented in Table 2.

As observed from Table 2, SVM displayed the weakest performance among all models, while ResNet, EfficientNet, and the proposed model in this study demonstrated superior capabilities, with the latter showing comparable performance to ResNet. The underlying reasons and mechanisms for these outcomes merit in-depth exploration. Firstly, SVM, a traditional machine learning method, often faces challenges when handling high-dimensional data like images. Furthermore, SVM heavily relies on the appropriate selection of kernel functions for classification, which might be limited when confronted with complex image features. In contrast, deep learning models such as ResNet and EfficientNet inherently possess the capability for automatic feature extraction, enabling them to discern distinctive features directly from raw images, leading to enhanced classification outcomes. ResNet, characterized as a deep residual network, introduces residual structures to address the vanishing gradient problem in deep networks, allowing for deeper training and richer feature representation. This is a primary reason for its commendable performance in this experiment. EfficientNet, on the other hand, employs model scaling strategies, enhancing model depth, width, and resolution without altering its size. The underlying principle suggests that strategic scaling can equip the model to capture intricate image features more effectively, subsequently boosting classification accuracy. DenseNet is distinguished by its densely connected structure. Each layer’s output connects with all subsequent layers, ensuring that the network receives feature information from every layer, thereby reinforcing feature reuse and enhancing feature propagation throughout the network. However, its performance falls slightly short when compared to ResNet and EfficientNet, potentially due to its model structure’s adaptability for this specific task being inferior to the latter two. Inception, VGG, Xception, and AlexNet, being earlier deep learning models, achieved notable results in their time. Yet, in comparison to newer models, their performance is evidently inferior. This is primarily attributed to the ongoing advancements in research, where continuous introductions of novel model structures and training strategies have led to consistent performance enhancements in models.

### 4.2. Model Attention Visualization

**Figure 7:**
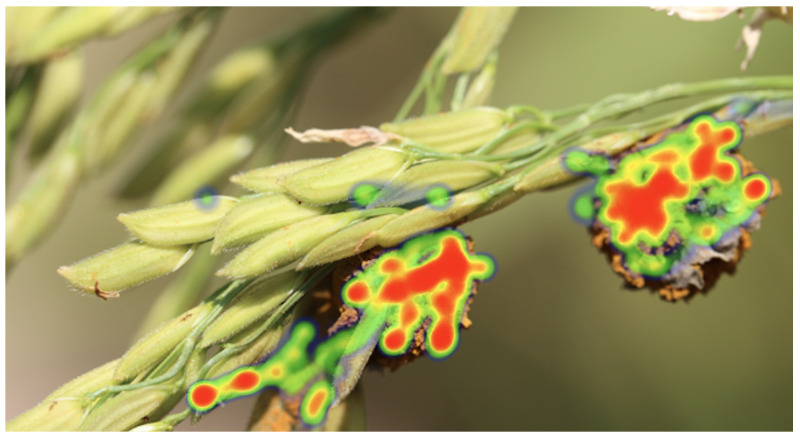
Visualization of model attention. The red region indicates the focal area of the model, while the green region signifies the nonfocal area of the model.

The primary objective of this section is to further validate the effectiveness and reliability of the proposed model. By visualizing attention points, it can be determined not only where the model focuses during disease detection but also whether these focus areas align with the actual disease regions. If the areas of attention of the model highly match the actual disease areas, it further attests to the model’s accuracy and reliability. The experimental results are shown in Figure 7.

Theoretically, deep learning models, especially convolutional neural networks, extract discriminative features from the original image through multiple layers of convolution. In the primary layers, the model might focus on basic image features, such as edges and textures, while in deeper layers, the model tends to concentrate on more complex features, such as the shape and structure of objects. In the model proposed in this paper, by introducing a model interpreter, the points of attention of the model are explicitly marked. These points represent the areas that the model deems most critical during the decision-making process. Hence, these focus points should primarily be concentrated in the disease regions. Analyzing from a mathematical perspective, the areas the model focuses on are related to the loss function used, optimization strategy, and model structure. In the proposed model of this paper, due to the adoption of a specific loss function and optimization strategy, the model is guided to focus on areas critical for classification decisions. Furthermore, the model’s structure is designed to effectively capture local features in the image, making the points of focus more concentrated and distinct.

In summary, visualizing model attention provides a powerful tool for verifying and explaining the model’s decision-making process. By comparing the model’s focus points with the actual disease regions, the accuracy and reliability of the model can be further validated. From a theoretical and mathematical standpoint, it can be understood why the model focuses on these specific areas and how these points of attention are associated with the model’s structure and optimization strategy.

### 4.3. Results on Transfer Learning

This section aims to investigate the performance of different models in the context of transfer learning. Transfer learning is a machine learning technique whose core idea is to transfer the knowledge of a model trained in one domain or task to another related domain or task. Specifically, transfer learning aims to accelerate and enhance the learning process of a new task by leveraging an existing pretrained model (usually trained on a large dataset). In the context of this paper, transfer learning is primarily employed for rice disease detection. Initially, a model pretrained on a large image dataset, such as ImageNet, is selected as the base model. This pretrained model is then fine-tuned to adapt to the new task of rice disease detection. Adopting this approach offers several benefits:

1. Accelerated learning: Since the base model has already learned some generic features and patterns, the fine-tuning process is typically much faster than training a model from scratch.
2. Improved performance: The pretrained model, often trained on a large dataset, can capture more complex features, contributing to enhanced performance on the new task.
3. Data efficiency: In certain cases, the target task, such as rice disease detection, might not have enough labeled data to train an efficient model from scratch. Using transfer learning allows for more effective utilization of limited data.
4. Generalization: As pretrained models are usually trained on more diverse datasets, they tend to have stronger generalization capabilities.

Through this approach, transfer learning in the context of this paper not only accelerates the model training process but also improves the model’s performance and generalization ability. By comparing the influence of various pretrained datasets on model performance, a deeper understanding of the value and effectiveness of transfer learning in agricultural disease detection can be obtained, as demonstrated in Table 3, Table 4 and Table 5.

Initially, the performance of the three models without any pretrained datasets is observed. Regardless of the model—whether it be the proposed model, ResNet, or EfficientNet—all display relatively lower accuracy, precision, and recall. This further underscores the performance-enhancing role of pretraining. Pretrained models can capture fundamental features and patterns in the data, thereby providing a more favorable starting point for new tasks. Subsequently, when ImageNet is employed as the pretraining dataset, a performance enhancement is noted across all models. ImageNet, a comprehensive image dataset, encompasses a plethora of features and patterns. This knowledge aids models, especially deep learning ones, in better understanding and identifying features within agricultural images. This pretrained knowledge can significantly expedite the convergence speed of the model and enhance its performance. Moreover, both the Wheat Competition and Plantsdoc datasets are pertinent to agriculture, making their role as pretrained datasets more pronounced in augmenting model performance. This aspect is evident across all models, especially in the proposed model, which exhibits the highest performance. This further validates the importance of the similarity between the pretrained dataset and the target task for the efficacy of transfer learning. From a structural perspective, the proposed model, ResNet, and EfficientNet are all rooted in deep convolutional neural networks. ResNet, by introducing a residual structure, mitigates the gradient vanishing issue in deep networks, enabling it to learn deeper features. EfficientNet, on the other hand, enhances the model’s performance without increasing computational demand through model expansion strategies. The proposed model, by integrating multisource data and transfer learning, further elevates its generalization capacity and accuracy.

In summary, transfer learning manifests immense potential and value in agricultural disease detection. Through judicious selection of pretrained datasets and model structural adjustments, the model’s performance can be further optimized to better cater to the unique characteristics and demands of agricultural scenarios.

### 4.4. Test on Multisource Data

The primary objective of the experiment design in this section is to validate the impact of multisource data on model performance, particularly in the specific task of rice disease detection. The efficacy and necessity of multisource data are demonstrated by comparing the performance of models trained solely on image data with those trained on multisource data, potentially comprising images, soil information, climate data, etc. The experiment results are shown in Table 6.

Firstly, a noticeable improvement in precision, recall, and accuracy is observed when multisource data are employed for training the models. Specifically, the precision increases from 0.87 to 0.94, and accuracy rises from 0.85 to 0.93, strongly suggesting that multisource data effectively enhance model performance. The reasons for such experimental results are:

1. Data diversity: Multisource data offer richer information than single-source image data. For instance, soil pH, temperature, and humidity may affect the occurrence and spread of diseases. When integrated with image data for training, the model can learn more features relevant to the target task of disease detection, thereby enhancing its performance.
2. Data complementarity: In certain scenarios, image data may have limitations, such as in low lighting or when subjects are obscured. Multisource data can offer a multifaceted perspective, compensating for the shortcomings of a single data source.
3. Reduced risk of overfitting: Utilizing multiple data sources implies that the model has to learn a wider range of feature combinations, which aids in enhancing the model’s generalization ability and reducing the risk of overfitting.
4. Strengthened decision making: Multisource data can offer comprehensive information, assisting the model in making more accurate and reliable decisions. For example, the model can judge diseases based not only on the color and shape of leaves but also by incorporating soil and climate information for a more holistic assessment.

From a mathematical standpoint, multisource data essentially expand the feature space of the model’s input. Assume that the original image data can be represented as a vector $X_{\mathrm{image}}$, and other types of data (e.g., soil information, climate data) can be represented as another vector $X_{\mathrm{other}}$. When multisource data are used, the model’s input effectively becomes $X=[X_{\mathrm{image}},X_{\mathrm{other}}]$, and this combined input vector can capture more useful information, thereby improving the model’s decision-making accuracy.

In summary, multisource data demonstrate significant advantages in the task of rice disease detection, not only elevating a model’s accuracy but potentially also enhancing its generalization ability and decision-making reliability. This confirms the effectiveness and reliability of the proposed interpretable high-accuracy rice disease detection method based on multisource data and transfer learning.

### 4.5. Test on Other Dataset

The primary aim of the experimental design is to assess the generalizability and adaptability of the proposed model across diverse datasets. By conducting tests on alternative datasets, an evaluation is undertaken to determine the model’s adaptability to different types of crops and diseases, as well as its performance under varying environmental conditions. The results are presented in Table 7.

Initially, it is evident from the results that the model demonstrates exceptional performance in terms of precision, recall, and accuracy, irrespective of whether the dataset pertains to wheat diseases or the Plantsdoc database. Such data strongly substantiate the model’s high degree of generalizability and adaptability. Theoretically, several factors may contribute to the model’s outstanding performance across multiple datasets:

1. Robust feature learning: The model possesses a highly complex structure and a large parameter space, enabling it to learn useful features and patterns from a variety of data. This is particularly crucial in the intricate and diverse field of agriculture.
2. Data preprocessing and augmentation: Techniques for data augmentation and preprocessing are employed during the model’s training phase. These not only enhance the model’s understanding of the raw data but also improve its generalization capabilities. Therefore, the model adapts more effectively to new, unseen data.
3. Advanced optimization algorithms: Sophisticated optimization algorithms and regularization techniques are utilized, aiding the model’s generalization across different datasets.

From a mathematical perspective, the model’s commendable performance could be attributed to its ability to effectively classify in high-dimensional feature spaces. It is hypothesized that the model learns a decision boundary during training, which mathematically could be represented as a complex multidimensional surface. If this boundary adapts well to the distribution of new data, then the model is likely to perform excellently. Moreover, if the training and test datasets share certain similarities or underlying features, the model is expected to perform well on the new datasets as well. In summary, the experimental results indicate that the proposed model exhibits strong generalizability and adaptability across different crops and disease conditions, validating its potential as an efficient and reliable tool for crop disease detection. Such characteristics broaden the model’s applicability, extending beyond specific types of crops or diseases to a wider range of agricultural environments and conditions.

### 4.6. Test on Dataset Augmentation

The primary objective of the experiment design is to investigate the impact of data augmentation on model performance, particularly within the specific application of rice disease detection. The experiment reveals the effect of various data augmentation strategies (including basic augmentation, Mixup, and Cutout) on the model’s precision, recall, and accuracy, as shown in Table 8.

Firstly, it is evident from the experimental results that models utilizing data augmentation strategies demonstrate marked improvements across all evaluation metrics. Notably, the highest precision, recall, and accuracy are observed when basic augmentation is combined with Mixup, strongly confirming the efficacy of data augmentation in enhancing model performance. Several key factors likely contribute to these outcomes: (1) Increased data diversity: Data augmentation generates additional training samples by applying various transformations (such as rotation, scaling, cropping, etc.) to the original data, thereby enriching data diversity. This enables the model to learn more features relevant to the target task, such as disease detection. (2) Reduced risk of overfitting: The augmentation increases the size and diversity of the training dataset, thereby potentially improving the model’s generalization capabilities and reducing the risk of overfitting. (3) Enhanced decision-making capabilities: With more comprehensive and diverse information provided through data augmentation, the model exhibits higher reliability and accuracy in decision making.

From a mathematical perspective, data augmentation is effectively manipulating the data distribution. The original dataset *D* can be considered as a sample drawn from a distribution *P*. Through data augmentation, a new dataset $D^{\prime }$ is obtained, representing samples from an expanded or adjusted distribution $P^{\prime }$. This allows the model to learn from a richer and more diverse data distribution during training. In summary, data augmentation exhibits significant advantages in rice disease detection, not only improving the model’s accuracy but also potentially enhancing its generalization abilities and decision-making reliability.

### 4.7. Discussion on Model’s Interpretation

In the present study, special emphasis is placed on the interpretability of the model, as this serves not only to elucidate the operational principles of the model for researchers but also fosters trust among agricultural experts and farmers, thereby facilitating its application in real-world settings. To delve deeper into the model’s interpretability, visualization of feature maps is employed. Feature maps are integral components of convolutional neural networks (CNNs), generated across various layers of the network and representing the model’s understanding of input data at different levels. Observing these feature maps enables a more intuitive understanding of how the model extracts useful information from complex input data, and how this information flows through the network to inform final decision making.

**Figure 8:**
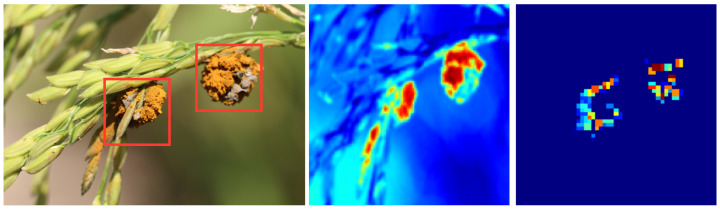
Visualization of feature maps in our model.

As illustrated in Figure 8, certain feature maps prominently highlight areas of disease spots on leaves, which are commonly critical indicators of the presence of disease. These observations align closely with the expert descriptions of the characteristics of rice diseases, thereby substantiating the model’s effectiveness and reliability. Furthermore, the feature maps allow observation of how information from multisource data, such as meteorological and soil data, is effectively fused by the model. These data sources often reflect different patterns or highlighted areas in distinct feature maps, yet they are synthesized for consideration in the final decision layer, leading to more accurate disease detection. The visualization of feature maps not only enhances the interpretability of the model but also provides an intuitive and effective means to understand and evaluate its performance in the task of rice disease detection. These observations not only affirm the high reliability and accuracy of the method proposed in this study but also further promote its broad application in agricultural production.

### 4.8. Limits and Feature Works

In this paper, a high-precision rice disease detection method based on multisource data and transfer learning that is interpretable is proposed. Although this method demonstrates superior performance in experiments, there are still some limitations, which in turn suggest directions for future research.

Initially, while the model’s generalization ability is verified across multiple datasets, it remains uncertain if it can adapt to all agricultural scenarios. Specific climate, soil, and other environmental factors might affect the growth conditions of rice, presenting challenges to the model’s detection capability. Hence, it is essential to consider further verifying the model’s generalization performance in more real-world scenarios. Additionally, although the model in this paper fully utilizes the advantages of multisource data, determining how to integrate this data more efficiently and intelligently remains an area worthy of exploration. The current data fusion strategy might not be optimal, and more advanced fusion algorithms could further enhance model performance. Moreover, the computational complexity of the model is a concern. High-precision models often come with high computational costs, potentially limiting their application in resource-constrained environments, such as mobile devices or onsite agricultural robots. For future endeavors, several directions are worth exploring:

1. Model optimization: Consider further optimizing the model structure to reduce its computational complexity while maintaining or enhancing its performance. Employing techniques like knowledge distillation or model pruning could be effective.
2. Smarter data fusion: Investigate advanced data fusion strategies, enabling the model to more effectively utilize information from different data sources.
3. Practical application and deployment: Contemplate deploying and verifying the model in more real-world agricultural scenarios, especially in resource-constrained settings like onsite fields or agricultural drones.

## 5. Conclusions

With the rapid advancement of agricultural modernization and precision agriculture, efficiently and accurately detecting and preventing crop diseases using advanced technological means has become a central topic in agricultural research. In this study, a method for interpretable high-precision rice disease detection based on multisource data and transfer learning was proposed, aiming to provide novel insights and tools for agricultural research and technological development. At the heart of this research is the accurate detection of rice diseases in the context of multisource data. Multisource data provide the model with richer and more diverse information, aiding in enhancing the model’s detection accuracy. Especially in complex agricultural environments, relying solely on a single data source often yields unsatisfactory results. By integrating a variety of data, such as images, climate, and soil, the model can gain a more comprehensive and in-depth understanding of disease conditions, leading to more accurate judgments. Furthermore, transfer learning endows the model with powerful generalization capabilities. Agricultural environments vary greatly due to factors such as region, climate, and season. Transfer learning allows the model to swiftly apply knowledge learned in one area or environment to another, significantly accelerating model deployment and reducing the cost of retraining. Additionally, the interpretability of the model is a major highlight of this study. In practical applications, model decisions often need to gain the trust and acceptance of farmers or agricultural experts. Through visualization of the model’s focus points, not only can the basis of the model’s decisions be clearly seen, but the accuracy and reliability of the model can also be further verified, providing strong support for its promotion and application.

In the experimental section, various advanced deep learning models were compared, validating the superior performance of the proposed method across multiple datasets. Particularly when compared to traditional machine learning methods, the method presented in this study exhibited significant advantages in core metrics such as precision, recall, and accuracy. This further attests to the immense potential and value of deep learning in agricultural disease detection. In summary, this paper offers a fresh perspective and approach to agricultural disease detection, combining the advantages of multisource data, transfer learning, and model interpretability, and contributes positively to the evolution of agricultural modernization and precision agriculture. In the future, it is hoped that the method proposed in this paper can be applied in more agricultural scenarios, bringing tangible benefits to farmers and agricultural researchers.

## Figures and Tables

**Table 1:** Distribution of image dataset used in this paper.

Disease Category	Number of Images
White leaf blight	218
Brown spot disease	187
Bacterial stripe disease	329
Sesame leaf spot disease	172
Rice aspergillosis disease	105
Rice blast disease	91
Healthy	1300

**Table 2:** Performance comparison of various models.

Model	Precision	Recall	Accuracy
SVM [22]	0.82	0.83	0.83
ResNet [23]	0.94	0.91	0.92
EfficientNet [28]	0.93	0.90	0.92
DenseNet [25]	0.91	0.89	0.90
Inception [24]	0.89	0.88	0.88
VGG [26]	0.88	0.87	0.87
Xception [29]	0.92	0.90	0.91
AlexNet [21]	0.87	0.86	0.86
Ours	0.94	0.92	0.93

**Table 3:** Results of our method on different transfer learning datasets.

Source Dataset	Precision	Recall	Accuracy
None	0.83	0.81	0.81
ImageNet [21]	0.88	0.91	0.90
Wheat Competetion [30]	0.91	0.90	0.91
Plantsdoc [31]	0.94	0.92	0.93

**Table 4:** Results of ResNet [23] on different transfer learning datasets.

Source Dataset	Precision	Recall	Accuracy
None	0.79	0.78	0.78
ImageNet [21]	0.83	0.87	0.85
Wheat Competetion [30]	0.90	0.92	0.90
Plantsdoc [31]	0.94	0.91	0.92

**Table 5:** Results of EfficientNet [27] on different transfer learning datasets.

Source Dataset	Precision	Recall	Accuracy
None	0.81	0.79	0.80
ImageNet [21]	0.84	0.81	0.83
Wheat Competetion [30]	0.87	0.90	0.89
Plantsdoc [31]	0.93	0.90	0.92

**Table 6:** Results of our method with(out) multisource data.

Data	Precision	Recall	Accuracy
Image data only	0.87	0.84	0.85
Multisource data	0.94	0.92	0.93

**Table 7:** Results of our method on other datasets.

Dataset	Precision	Recall	Accuracy
Wheat Diesease	0.95	0.97	0.95
Plantsdoc [31]	0.96	0.97	0.97

**Table 8:** Results of our method with(out) dataset augmentation.

Data	Precision	Recall	Accuracy
None	0.86	0.87	0.86
Basic Augmentation	0.91	0.90	0.91
Basic Augmentation + Mixup [32]	0.94	0.92	0.93
Basic Augmentation + Cutout [33]	0.93	0.92	0.92
